# Epidemiological study of unusual rotavirus strains and molecular characterization of emerging P[14] strains isolated from children with acute gastroenteritis during a 15-year period

**DOI:** 10.1007/s00705-023-05769-8

**Published:** 2023-05-02

**Authors:** Elizabeth-Barbara Tatsi, Dimitra-Maria Koukou, Charilaos Dellis, Maria-Myrto Dourdouna, Vasiliki Efthymiou, Athanasios Michos, Vasiliki Syriopoulou

**Affiliations:** 1 First Department of Pediatrics, Infectious Diseases and Chemotherapy Research Laboratory, Medical School, National and Kapodistrian University of Athens, “Aghia Sophia” Children’s Hospital, Athens, 11527 Greece; 2 University Research Institute of Maternal and Child Health and Precision Medicine, Athens, Greece

**Keywords:** Rotavirus, Children, Gastroenteritis, Unusual genotype, Molecular characterization, Phylogenetics

## Abstract

Rotavirus group A (RVA) is characterized by molecular and epidemiological diversity. To date, 42 G and 58 P RVA genotypes have been identified, some of which, like P[14], have a zoonotic origin. In this study, we describe the epidemiology of unusual RVA genotypes and the molecular characteristics of P[14] strains. Fecal samples from children ≤ 16 years of age with acute gastroenteritis (AGE) who were hospitalized during 2007–2021 in Greece were tested for RVA by immunochromatography. Positive RVA samples were G and P genotyped, and part of the VP7 and VP4 genes were sequenced by the Sanger method. Epidemiological data were also recorded. Phylogenetic analysis of P[14] was performed using MEGA 11 software. Sixty-two (1.4%) out of 4427 children with RVA AGE were infected with an unusual G (G6/G8/G10) or P (P[6]/P[9]/P[10]/P[11]/P[14]) genotype. Their median (IQR) age was 18.7 (37.3) months, and 67.7% (42/62) were males. None of the children were vaccinated against RVA. P[9] (28/62; 45.2%) was the most common unusual genotype, followed by P[14] (12/62; 19.4%). In the last two years, during the period of the COVID-19 pandemic, an emergence of P[14] was observed (5/12, 41.6%) after an 8-year absence. The highest prevalence of P[14] infection was seen in the spring (91.7%). The combinations G8P[14] (41.7%), G6P[14] (41.7%), and G4P[14] (16.6%) were also detected. Phylogenetic analysis showed a potential evolutionary relationship of three human RVA P[14] strains to a fox strain from Croatia. These findings suggest a possible zoonotic origin of P[14] and interspecies transmission between nondomestic animals and humans, which may lead to new RVA genotypes with unknown severity.

## Introduction

Acute gastroenteritis (AGE) is characterized by the presence of diarrhea and/or vomiting, possibly accompanied by fever, abdominal pain, and dehydration [1]. It is responsible for approximately 10% of pediatric mortality worldwide [2]. Depending on the country, rotaviruses (RVs) are among the most common pathogens causing AGE [3, 4]. Although several groups of rotaviruses (A-D and F-J) have been identified, only A-C and H can infect both humans and animals. Among the human rotaviruses, rotavirus group A (RVA) is the most common [5, 6].

Neonates and children under the age of 5 years old are the most frequently infected with RVA, and according to reports of the Global Burden of Disease, RV infection causes > 250 million episodes of diarrhea and > 120 thousand deaths annually [7]. However, more than ten years after the implementation of the RVA vaccines Rotarix® (GlaxoSmithKline, Rixensart, Belgium, RV1) and RotaTeq® (Merck & Co, Lyon, France, RV5), a 40.6% decrease in the mortality rate has been observed in children < 5 years old [8–10].

RVA belongs to the family *Reoviridae*, and its genome consists of 11 double-stranded RNA segments, which encode six structural (VP1-VP4, VP6, and VP7) and six non-structural viral proteins (NSP1-NSP6) [11]. For the genotyping of RVA, an international classification system has been established based on the sequences of two viral proteins: VP4 (protease-sensitive protein) for the P genotype and VP7 (glycoprotein) for the G genotype [12]. These two proteins form the outer capsid of the virus, participate in host cell attachment and penetration, and also contain neutralization epitopes [13].

To date, at least 42 G and 58 P RVA genotypes have been identified in humans and animals (https://rega.kuleuven.be/cev/viralmetagenomics/virus-classification/rcwg). Several domestic animals such as dogs, cats, cows, and pigs as well as non-domestic animals such as bats, rodents, and birds can be infected with RVs [14]. Although more than 80 G and P combinations of RVA have been detected, the most common RVA genotypes circulating in humans worldwide are G1P[8], G2P[4], G3P[8], G4P[8], G9P[8], and G12P[8] [15]. Unusual genotypes such as P[14] and mixed types (with more than one G and/or P type) have also been reported. Strains carrying the unusual P[14] genotype are sporadically detected in humans and usually show a zoonotic origin [16]. It is not unprecedented that human-to-animal contact may contribute to interspecies transmission, as appears to be the case for P[14] strains [17].

The aims of this study were to describe the epidemiology of unusual RVA genotypes isolated from children presenting with AGE from 2007–2021 and to examine the molecular characteristics of human P[14] RVA strains.

## Materials and methods

### Study design

This was a multicenter study conducted at the Infectious Diseases Laboratory of the Choremeion Research Laboratory, “Aghia Sophia” Children’s Hospital, and involved the analysis of 4427 fecal samples from children ≤ 16 years old with AGE from 20 Greek paediatric hospitals during the period 01/2007–12/2021.

Children who were admitted to “Aghia Sophia” Children’s Hospital, a 750-bed tertiary paediatric hospital in the metropolitan Athens area, or to 19 other paediatric hospitals, covering the majority of the national paediatric population, and fulfilled the criteria for AGE were tested for rotavirus infection using a rapid immunochromatographic test (VIKIA® Rota-Adeno test, bioMérieux, Lyon, France). Samples that were positive for RVA were sent within a 10-day period to the Infectious Diseases Laboratory of the Choremeion Research Laboratory in Athens for RVA genotyping according to EuroRotaNet’s guidelines (https://www.eurorotanet.com/project-information/documents-and-methods/). The study protocol was approved by the scientific and bioethics committee of “Aghia Sophia” Children’s Hospital (No. 6261).

### Data collection

Demographic and clinical data were also collected from children infected with unusual RVA, including age, sex, residence, symptoms of AGE (diarrhea, vomiting, fever, and dehydration), laboratory data from blood samples, days of hospitalization, and RVA vaccination status. Laboratory data included values for potassium (K^+^), sodium (Na^+^), calcium (Ca^2+^), chlorine (Cl^-^), C-reactive protein (CRP), urea, creatinine, white blood cells (WBC), polymorphonuclear leukocytes, and lymphocytes that were requested in the context of hospitalization for AGE.

### Reverse transcription and gene amplification

Fecal samples were prepared as a 10% suspension with Stool Transport and Recovery (S.T.A.R.) buffer (Roche Diagnostics, Basel, Switzerland) and 7% chloroform for the extraction of viral genomic RNA, employing a MagNA Pure Compact Nucleic Acid Isolation Kit I (Roche Diagnostics, Basel, Switzerland) on a MagNA Pure Compact instrument according to the manufacturer’s instructions. Viral RNA was stored at -80°C or used immediately.

Synthesis of cDNA was carried out using a Transcriptor First Strand cDNA Synthesis Kit (Roche Diagnostics, Basel, Switzerland) according to the manufacturer’s instructions. Polymerase chain reaction (PCR) and multiplex semi-nested PCR for the amplification of the VP7 and VP4 regions were conducted using GoTaq DNA Polymerase (Promega, Madison, Wisconsin, USA) and specific primers according to European Rotavirus Detection and Typing Methods [18]. The PCR products were initially characterized as G (G1-4, G8-10, and G12) and P (P[4], P[6], P[8], and P[9]-[11]) types according to the PCR product size, using 2% agarose gel electrophoresis with a 50-bp DNA ladder (N3236S; New England Biolabs, Massachusetts, USA) and ethidium bromide staining.

### Sequencing

Further genotyping of RVA strains that were not genotyped by agarose gel electrophoresis was carried out by Sanger sequencing of the VP7 and VP4 genes using a BigDye Terminator v3.1 Cycle Sequencing Kit on an Applied Biosystems 3500 Genetic Analyzer (Applied Biosystems, Waltham, MA, USA). The electrochromatographic data from sequencing were further analyzed using BLAST (https://blast.ncbi.nlm.nih.gov/Blast.cgi).

### Phylogenetic analysis

Phylogenetic evolutionary analysis of P[14] RVA strains was performed using MEGA 11 software (Molecular Evolutionary Genetics Analysis; www.megasoftware.net). All P[14] sequences from human and animal strains were obtained from the GenBank (https://www.ncbi.nlm.nih.gov/genbank/) and Virus Variation (https://www.ncbi.nlm.nih.gov/genome/viruses/variation/) databases. Multiple sequence alignments were performed using MUSCLE (Multiple Sequence Comparison by Log-Expectation) software. A model/method test was performed. Phylogenetic trees were constructed using the maximum-likelihood method, the Tamura 3-parameter model, and bootstrap resampling with 1000 replicates. The substitution model used was the maximum composite likelihood model. Nucleotide sequence similarity was analyzed using BLAST. The sequences determined in this study were deposited in the GenBank database with accession numbers ON009343-44, ON004940, OM912818-19, OM303087, OM891773, OM829817, OM303090, OP183207-08, and OQ606801.

## Results

### Genotyping and study population

In this study, 4427 fecal samples from children ≤ 16 years with AGE who were infected with an RVA strain were included. Genotyping showed that 62 (1.4%) children with a median age of 18.7 (IQR: 37.3) months had been infected by an unusual G (G6: 16.1%; G: 11.3%; G10: 4.8%) or P (P[6]: 17.7%; P[9]: 45.2%; P[10]: 9.7%; P[11]: 1.6%; P[14]: 19.4%) genotype. The majority of these children belonged to the age group of 13–48 months (25/62, 40.3%), 67.7% (42/62) were male, 25.8% resided in rural areas, and 63% (39/62) were of Greek origin. None of them had been vaccinated for RVA. Their AGE symptoms were diarrhea (77.4%; 48/62), vomiting (58.1%; 36/62), fever (66.1%; 41/62), and moderate dehydration (40.3%; 25/62). Their laboratory values were within the normal range, except for CRP, which was slightly elevated (mean ± SD: 17.2 ± 28.7). Their median (IQR) days of hospitalization was 3.0 (2.0) days.

In total, P[14] (19.4%; 12/62) was the second most common unusual genotype after P[9] (28/62; 45.2%). Three different genotype combinations with P[14] were detected, G8P[14] (41.7%; 5/12), G6P[14] (41.7%; 5/12), and G4P[14] (16.6%; 2/12). The genotype combinations of unusual RVA strains detected in this study are shown in Table 1. The median (IQR) age of children with P[14] RVA infection was 50.6 (52.12) months, 58.3% (7/12) were males, 83.4% (10/12) were of Greek origin, 8.3% (1/12) were of Afghan origin, 8.3% (1/12) were of Albanian origin, and 33.3% (4/12) resided in rural areas. Their symptoms included diarrhea (10/12; 83.3%), vomiting (8/12; 66.6%), fever (8/12; 66.6%), and moderate dehydration (7/12; 58.3%). Specifically, diarrhea or vomiting was present in 58.3% (7/12), and both symptoms were present in 50% (6/12) of the patients. The median (IQR) days of hospitalization was 3.33 (2.07).

**Table 1:** Detected G and P combinations in unusual rotavirus group A strains

G type n (%)	P type n (%)							Total
	P[6]	P[8]	P[9]	P[10]	P[11]	P[14]	P[UD]	n (%)
G1	-	-	1 (1.6)	-	-	-	-	1 (1.6)
G2	2 (3.2)	-	-	1 (1.6)	-	-	-	3 (4.8)
G3	-	-	14 (22.6)	-	-	-	-	14 (22.6)
G4	2 (3.2)	-	3 (4.8)	1 (1.6)	-	2 (3.2)	-	8 (12.9)
G6	-	-	4 (6.5)	-	-	5 (8.1)	-	9 (14.5)
G8	1 (1.6)	1 (1.6)	-	-	-	5 (8.1)	-	7 (11.3)
G9	-	-	4 (6.5)	4 (6.5)	-	-	-	8 (12.9)
G10	-	2 (3.2)	-	-	-	-	1 (1.6)	3 (4.8)
G12	6 (9.7)	-	1 (1.6)	-	1 (1.6)	-	-	8 (12.9)
G-UD	-	-	1 (1.6)	-	-	-	-	1 (1.6)
Total n (%)	11 (17.7)	3 (4.8)	28 (45.2)	6 (9.7)	1 (1.6)	12 (19.4)	1 (1.6)	62

UD, unidentified type

Regarding the laboratory values for children infected with P[14] RVA infection, it was found that all of the children had slightly elevated CRP (mean ± SD: 18.2 ± 19.1 mg/l; normal values, 0–1 mg/l for children ≤ 1 year old and 1–10 mg/l for those > 1 year old), and one child showed electrolytic disturbances with low potassium (K^+^: 3.4 mmol/l; normal values: 3.5–5.5 mmol/l) and sodium (Na^+^: 133 mmol/l; normal values, 135–150 mmol/l). All of the other values were normal.

### Annual and seasonal distribution

**Fig. 1:**
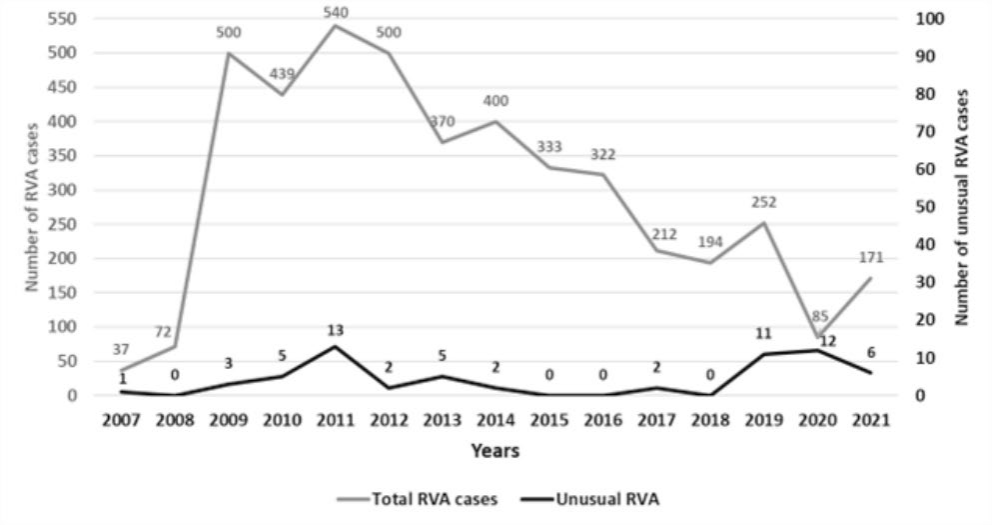
Annual distribution of total (n = 4427) and unusual (n = 62) rotavirus group A (RVA) cases from 2007–2021

An increase in the annual distribution of unusual RVA strains was observed in 2019–2021, in the COVID-19 pandemic period, with the highest circulating levels (14.1%; 12/85) in 2020 (Fig. 1). Genotypes with a prevalence > 1% were detected: P[6] (1.3%) in 2011, P[9] (1.7%) and P[10] (2%) in 2019, P[9] (9.4%), P[14] (2.4%), G8 (2,4%), and G10 (1.2%) in 2020, and P[9] (1.8%), P[14] (1.8%), and G6 (2.3%) in 2021. The seasonal peak was during spring (March to May: 37.1%; 23/62) and autumn (September to November: 33.9%; 21/62), whereas the prevalence was lower in winter (20.9%; 13/62) and summer (8.1%; 85/62).

Regarding P[14] strains, the annual distribution was as follows: 2.7% (1/37) in 2007, 0.9% (4/439) in 2010, 0.4% (2/500) in 2012, 2.4% (2/85) in 2020, and 1.8% (3/171) in 2021. In the last two consecutive years, during the COVID-19 pandemic period, an emergence of this strain was observed (1.6%; 4/256 in all RVA samples and 41.7%; 5/12 in P[14]) after 8 years of absence. Their seasonal peak occurred during the spring months, March to May (91.7%; 11/12). P[14] genotypes were detected in 12 children from six different children’s hospitals. The geographical distribution of this genotype in Greece was as follows: eight genotypes were isolated in Athens (three hospitals), two in Karditsa, one in Larisa (a previously reported genotype [19]), and one in Kalamata.

### Molecular characterization of P[14] strains

From the 12 samples from children with P[14] RVA infection, a portion of the VP4 gene was sequenced. This part includes almost all of the VP8* region (amino acids 1-231), the fragment between the VP8* and VP5* regions, and the start of the VP5* region (up to amino acid 264). The P[14] sequences of these strains were aligned and compared with all recorded P[14] RVA (human and animal) sequences (n = 94).

Many synonymous and missense gene variants were detected. Three of the missense variants were *novel*: V61I, Q62S, and Q127R. The variants V61I and Q62S, detected in RVA/Human-wt/GR/20210521/3005/2021/G8P[14] (OM303090.1) and RVA/Human-wt/GR/20210605/2974/2021/G6P[14] (OM829817.1), respectively, are in the VP8* region, which interacts with the viral protein VP6 [11] [20]. The mutation V61I involves the replacement of a hydrophobic amino acid with another one, and the mutation Q62S represents a replacement of a hydrophilic amino acid with a hydrophobic neutral amino acid. The *novel* VP4 variant Q127R was detected in the strain RVA/Human-wt/GR/20100409/517/2010/G6P[14] (OM912819.1). This variant is located in the VP8* spike head, in the lectin-like, globular domain, and involves the replacement of one hydrophilic amino acid with another hydrophilic amino acid.

When comparing the sequences of the P[14] RVA strains from this study with those of the antigenic epitopes of the VP8* region of the Rotarix and RotaTeq vaccine strains [21], many different variants were detected (Table 2). The amino acid D100 in the 8 − 1 epitope appears to be conserved, except in the strain RVA/Human-wt/GR/20120422/19–38/2012/G6P[14], which carries the homozygous D100N variant, which involves the replacement of a negatively charged amino acid, aspartic acid (D), with an uncharged amino acid, asparagine (N), which has a similar size. This position is probably important because it is next to amino acid 101, which binds to sialic acid on the host cell. A search of the other 94 recorded P[14] sequences showed that only three carried this variant: RVA/Human-wt/JPN/Tokyo/12-1375/2012/G8P[14], RVA/Human-wt/BEL/B4106/2000/G3P[14], and RVA/Human-wt/BEL/BE5028/2012/G3P[14]. The frequency of this variant among P[14] strains (n = 106) was thus estimated to be 3.8%.

**Table 2:** Variants e in antigenic epitopes of the VP8* region of P[14] rotavirus group A (RVA) strains (n = 12)

	8 − 1											8 − 2		8 − 3									8 − 4		
	100	146	148	150	188	190	192	193	194	195	196	180	183	113	114	115	116	125	131	132	133	135	87	88	89
Rotarix^™^	D	S	S	N	S	S	A	N	L	N	N	E	R	N	P	V	D	S	S	N	D	N	N	T	N
RotaTeq^™^	D	S	S	N	S	N	A	N	L	N	D	E	R	N	P	V	D	N	R	N	D	D	N	T	N
RVA/Human-wt/GR/20070101/057/2007/G8P[14]	D	**L**	**K**	**G**	**Y**	**L**	**I**	N	**N**	**D**	N	**T**	**N**	**S**	**N**	**T**	**Q**	**T**	S	N	D	**S**	**T**	**Q**	**I**
RVA/Human-wt/GR/20100331/50016/2010/G4P[14]	D	**L**	**K**	**G**	**Y**	**L**	**I**	N	**N**	**D**	N	**T**	**N**	**S**	**N**	**T**	**Q**	**T**	S	N	D	**S**	**T**	**Q**	**I**
RVA/Human-wt/GR/20100406/5–12/2010/G4P[14]	D	**L**	**K**	**G**	**Y**	**L**	**I**	N	**N**	**D**	N	**T/I**	**N**	**S**	**N**	**T**	**Q**	**T**	S	N	D	**S**	**T**	**Q**	**I**
RVA/Human-wt/GR/20100407/60036/2010/G6P[14]	D	**L**	**K**	**G**	**Y**	**L**	**I**	N	**N**	**D**	N	**T**	**N**	**S**	**N**	**T**	**Q**	**T**	S	N	D	**S**	**T**	**Q**	**I**
RVA/Human-wt/GR/20100409/517/2010/G6P[14]	D	**L**	**K**	**G**	**Y**	**L**	**I**	N	**N**	**D**	N	**T**	**N**	**S**	**N**	**T**	**Q**	**T**	S	N	D	**S**	**T**	**Q**	**I**
RVA/Human-wt/GR/20120417/1100/2012/G8P[14]	D	**L**	**K**	**G**	**Y**	**L**	**I**	N	**N**	**D**	N	**T**	**N**	**S**	**N**	**T**	**Q**	**T**	S	N	D	**S**	**T**	**Q**	**I**
RVA/Human-wt/GR/20120422/19–38/2012/G6P[14]	**N**	**L**	**K**	**G**	**Y**	**L**	**I**	N	**N**	**D**	N	**T**	**N**	**S**	**N**	**T**	**Q**	**T**	S	N	D	**S**	**T**	**Q**	**I**
RVA/Human-wt/GR/20200422/2887/2020/G8P[14]	D	**L**	**K**	**G**	**Y**	**L**	**I**	N	**N**	**D**	N	**T**	**N**	**Q**	**N**	**T**	**Q**	**A**	S	N	D	**S**	**T**	**Q**	**I**
RVA/Human-wt/GR/20200509/2885/2020/G8P[14]	D	**L**	**K**	**G**	**Y**	**L**	**I**	N	**N**	**D**	N	**T**	**N**	**S**	**N**	**T**	**Q**	**T**	S	N	D	**S**	**T**	**Q**	**I**
RVA/Human-wt/GR/20210605/2974/2021/G6P[14]	D	**L**	**K**	**G**	**Y**	**L**	**I**	N	**N**	**D**	N	**T**	**N**	**S**	**N**	**T**	**Q**	**T**	S	N	D	**S**	**T**	**Q**	**I**
RVA/Human-wt/GR/20210521/3005/2021/G8P[14]	D	**L**	**K**	**G**	**Y**	**L**	**I**	N	**N**	**D**	N	**T**	**N**	**S**	**N**	**T**	**Q**	**T**	S	N	D	**S/G**	**T**	**Q**	**I**
RVA/Human-wt/GR/20210516/3096/2021/G6P[14]	D	**L**	**K**	**G**	**Y**	**L**	**I**	N	**N**	**D**	N	**T**	**D**	**P**	**N**	**T**	**Q**	**T**	S	N	**N**	**S**	**T**	**Q**	**T**

Rotarix^™^ = G1P[8] A41CB052A; RotaTeq^™^ = G6P[8] WI79-4

The strain RVA/Human-wt/GR/20210516/3096/2021/G6P[14] also carried three variants when compared to the vaccine strains and the other strains from this study: N/I89T, N/S113P, and R/N183D (Table 2). Although these amino acid positions are probably less conserved, none of them are *novel*. The variant N/I89T is in the 8 − 4 epitope, and it was previously reported in three animal strains (rabbit and antelope) and five human strains in Europe and North America from 1989. The variant N/S113P is located within the 8 − 3 epitope and has previously been reported only in two animal strains from America (RVA/Guanaco-wt/ARG/Chubut/1999/G8P14) and Africa (RVA/Goat-wt/MAR/Ch_S44/2014/P14). The variant R/N183D, located in the 8 − 2 epitope, was found in three human strains from Europe (RVA/Human-wt/ITA/PA77/2002/P14, and RVA/Human-wt/ESP/Sp813/2007/P14, RVA/Human-wt/BEL/B10925/1997/G6P14). The frequency of these variants among the P[14] strains (n = 106) was 8.5%, 2.8%, and 3.8%, respectively.

Position 113 seems to be variable, since one of our 12 P[14] strains RVA/Human-wt/GR/20200422/2887/2020/G8P14, carried the variant N/S113Q, which is also present in 27 animal and human P[14] strains (frequency: 26.4%). Despite the physicochemical differences between all these amino acids, their contribution to transmissibility and morbidity remains unknown.

### Phylogenetic analysis of P[14] strains

**Fig. 2:**
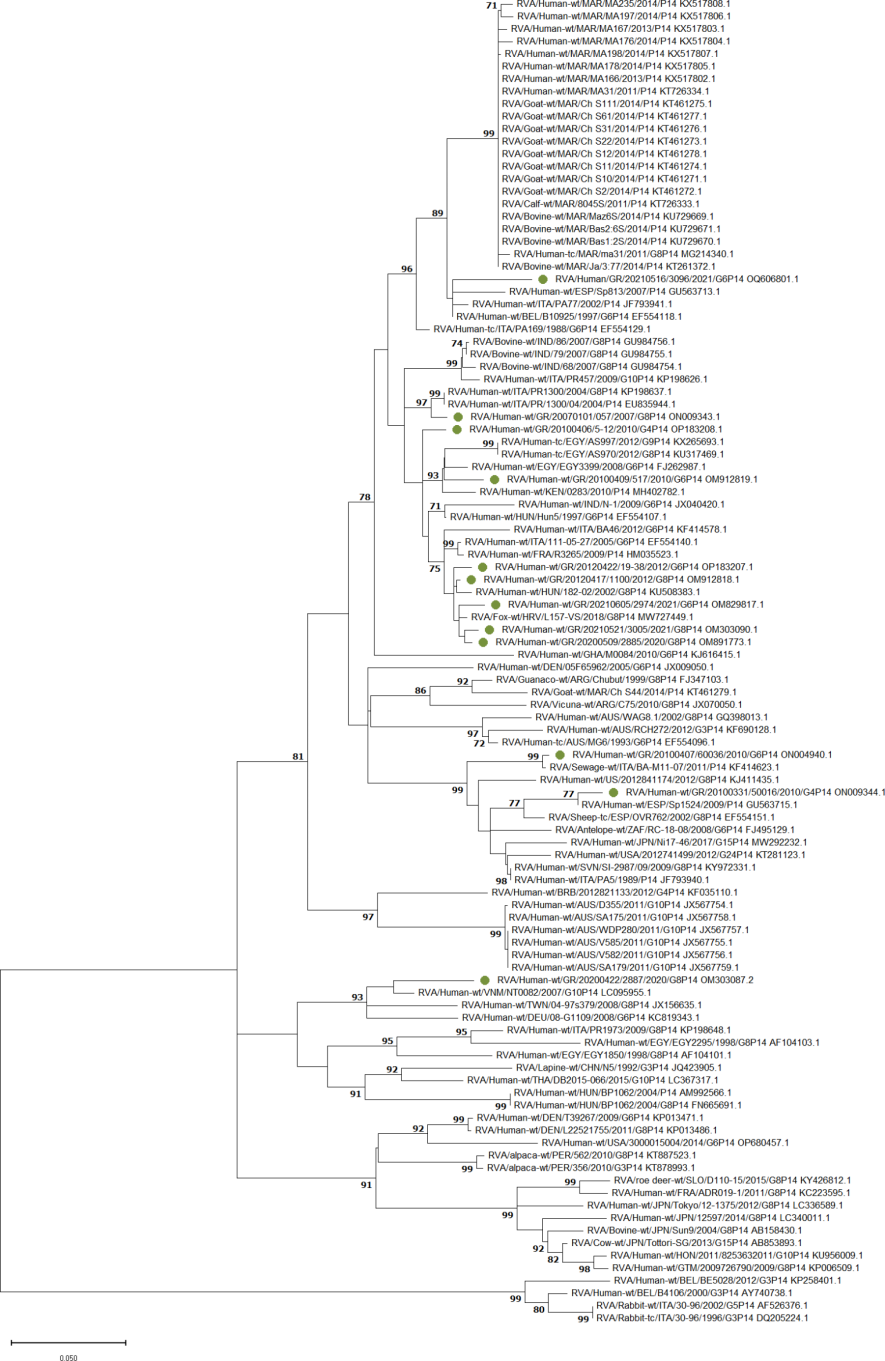
Phylogenetic tree of P[14] rotavirus group A (RVA) strains based on partial VP4 gene sequences. Samples from this study are indicated by a green circle. Sequences obtained from the GenBank database are labeled with their accession number, two or three letters of the country of origin, the year of isolation, and their genotype. The maximum-likelihood method, the Tamura 3-parameter model, and a bootstrap test of 1000 replicates were used to infer the phylogeny. Bootstrap values above 70% are shown next to branches. The bar represents 0.050 substitutions per nucleotide position. HRV, Croatia; HUN: Hungary; ITA: Italy; FRA: France; KEN: Kenya; EGY: Egypt; IND: India; ESP: Spain; BEL: Belgium; MAR: Morocco; GHA: Ghana; ARG: Argentina; AUS: Australia; USA: United States of America; JPN: Japan; TUN:Tunisia; BRB: Barbados; THA: Thailand; DEU and GER: Germany; TWN: Taiwan; VNM: Vietnam; PER: Peru; GTM: Guatemala; HON: Honduras; GR: Greece; ZAF: South Africa; CHN: China, SLO and SVN: Slovenia

Phylogenetic analysis was performed using 106 VP4 gene sequences (94 from public databases and 12 from this study) to clarify their genetic relationships and evolutionary history (Fig. 2). Nine of the strains from this study may have a European ancestor, three of which probably originated from an RVA strain that was detected in a wild animal. One may have originated in Egypt, another in Vietnam, and another in either Hungary or India.

Phylogenetics showed that the VP4 amino acid sequence of strain RVA/Human-wt/GR/20070101/057/2007/G8P[14] is 97% related to that of the 2004 Italian strains RVA/Human-wt/ITA/PR1300/2004/G8P[14] and RVA/Human-wt/ITA/PR/1300/04/2004/P[14], with 99% nucleotide sequence identity due to the presence of seven synonymous and two missense mutations as shown using BLAST. The strain RVA/Human-wt/GR/20100331/50016/2010/G4P[14] was 77% related to the Spanish strain RVA/Human-wt/ESP/Sp1524/2009/P[14], with 99% nucleotide sequence identity (five synonymous and one missense mutation). Both strains also were 77% related to a farm animal strain from Spain, RVA/Sheep-tc/ESP/OVR762/2002/G8P[14]. The VP4 of strain RVA/Human-wt/GR/20100406/5–12/2010/G4P[14] is less than < 70% related to other strains.

The VP4 of Greek strain RVA/Human-wt/GR/20100407/60036/2010/G6P[14] is 99% evolutionary related to that of Italian strain RVA/Sewage-wt/ITA/BA-M11-07/2011/P[14], which was isolated from sewage in 2011, with only three variants (also 99% nucleotide identity), all of which are synonymous substitutions. The ancestor of RVA/Human-wt/GR/20100409/517/2010/G6P[14] may be (<70% bootstrap value) a strain from Egypt in 2008 (RVA/Human-wt/EGY/EGY3399/2008/G6P[14]), as these strains share 70% nucleotide sequence identity. Phylogenetic analysis showed that the strains RVA/Human-wt/GR/20200509/2885/2020/G8P[14], RVA/Human-wt/GR/20210605/2974/2021/G6P[14], and RVA/Human-wt/GR/20210521/3005/2021/G8P[14] formed a subcluster with the MW727449.1 red fox strain RVA/Fox-wt/HRV/L157-VS/2018/G8P[14] from Croatia in 2018, with a bootstrap value < 70% and 99% nucleotide identity. This subcluster belongs to a cluster with bootstrap probability of < 70% that includes the strains RVA/Human-wt/HUN/182-02/2002/G8P[14], RVA/Human-wt/GR/20120422/19–38/2012/G6P[14], and RVA/Human-wt/GR/20120417/1100/2012/G8P[14], together with the fox strain (99% nucleotide sequence identity).

The strain RVA/Human-wt/GR/20200422/2887/2020/G8P[14] was found to be distantly related to the other samples from our study and showed less than 70% relation to the human strain RVA/Human-wt/VNM/NT0082/2007/G10P[14] from Vietnam in 2007, with 95% nucleotide sequence identity. The strain RVA/Human-wt/GR/20210516/3096/2021/G6P[14] was also found to be distantly related (< 70%) to other European strains, with 95–96% nucleotide sequence identity.

## Discussion

In this study, we investigated the epidemiology of unusual RVA strains detected in 62 hospitalized children with rotavirus AGE from 2007 to 2021 in Greece and performed a genetic and phylogenetic analysis of P[14] strains. In recent years, an increase in the circulation of unusual RVA strains has been detected. Multiple variants were detected in the antigenic epitopes of the VP8* region, but their significance is unknown. A distant evolutionary relationship was found between Greek human P[14] RVA strains and a Croatian fox P[14] RVA strain, which suggests a possible zoonotic origin of P[14] and interspecies transmission from a wild animal to humans.

Data obtained before the implementation of the RVA vaccine demonstrate that the genotypes P[6], P[9], and P[11] circulated at a significantly higher rate in America, Africa, and Asia than in Europe and Australia, while G8 mainly circulated in Africa [22]. The implementation of vaccines has an impact on the genotype distribution, resulting in the detection of a greater variety of genotypes around the world, with an increase in G9 and G12 as well as in unusual genotypes [23, 24]. Changes in genotype distribution were also observed during the COVID-19 pandemic. In Australia, from 2020 to 2021, a remarkable increase in G8P[8] from 1–87.5% was reported, with a peak in autumn, while the prevalence of G8P[14] decreased from 7.1% to < 1% [25, 26]. In a longitudinal study from 2014 to 2020 in Japan, the frequency of G8P[8] increased from 34% in 2017–2018 to 98% in 2018–2019, peaking in the spring [27]. In Europe, the genotypes G8P[8] and G8P[14] were detected at a prevalence of > 1%, in contrast to previous years [28]. Similarly, in our study, in the period 2019–2021, many different unusual genotypes were detected at > 1% prevalence, including P[9] and P[10] in 2019, P[9], P[14], G8, and G10 in 2020, and P[9], P[14], and G6 in 2021, with a seasonal peak in the spring.

P[14] is an unusual genotype, and its frequency differs in different countries. In our 15-year study, its frequency was < 1%. Similar to what has been observed in other countries, P[14] was mostly found together with genotype G8 in our study. In Venezuela, before the implementation of an RVA vaccine, the G8P[14] was the sixth most common RVA genotype, but after the start of vaccination, G8P[14] (2.4%) was the third most common RVA genotype detected, after G2P[4] and G1P[8] [29, 30]. In Japan, only one out of 247 RVA-positive samples had the G8P[14] combination. This sample was detected in the winter of 2014 [31], in contrast to our P[14] samples, which were mostly detected in the spring.

In this study, in addition to G8, the P[14] genotype was also combined with G6 and G4. Genotypes G6 and G8, have been detected sporadically (< 1%) in humans in previous studies, suggesting zoonotic origin [32–34], primarily from bovines [35, 36]. These genotypes are more common in countries that do not follow strict hygiene measures and people come into contact very often with farm animals as well as wildlife [37].

Although G8 and G6 are common in bovines [22], G8 is also found in oysters and shellfish, although it does not infect them [38]. In Bangladesh, the predominant genotype (> 94%) among 200 diarrheic calves was G6P[11] [39], while during 2017–2019 in India, G6P[14] and G8P[14] were the most common RVA genotypes in bovines [40]. In Brazil, in 2010–2016, only 3% of children < 3 years old with AGE were infected with G8P[4] [41], while in West Africa, in 2010, an infant with diarrhea was infected with RVA G6P[14] [42]. In a study in Pakistan in 2015 that included 180 samples from children < 5 years old with AGE, G8 was among the most common RVA strains in infants [43], but in a study in Iran in the period of 2017–2019, G8, G6, and P[14] were not detected in any of 130 children < 15 years old with AGE [44]. Also, G8 strains were reported by EuroRotaNet as significant emerging strains in the UK during 2008–2010 and 2018–2019 (EuroRotaNet, annual report 2019).

Recently, a new combination, G9P[14], was detected in Egypt in a 6-month-old child with AGE [45]. The sequence of P[14] in this strain was very similar to the old sequence EF554107 (95.45%) from Hungary [45]. This probably indicates that P[14] has been conserved over time.

Previous phylogenetic and genetic analysis of RVA strains from feces of humans and animals with gastroenteritis has suggested that P[14] has a zoonotic origin, most likely from bovines. A study from Japan showed that P[14] sequences from humans were similar to sequences present in bovine strains [31]. In this study, we found a distant relationship between human P[14] RVA and fox P[14] RVA, with a bootstrap value < 70%. This finding suggests the interspecies transmission between wild animals and humans.

So far, there is only one study from Croatia, where foxes live near urban areas, that refers to RVA infection in fox feces. It was shown that one red fox (*Vulpes vulpes*) was infected with a G8P[14] RVA strain in 2018 [46]. Therefore, wild animals such as foxes may be potential reservoirs for unusual strains.

The use of next generation sequencing (NGS) can help to elucidate possible reassortment events, the number of these events, and the potential zoonotic origin of a strain. Studies from various countries have demonstrated the susceptibility of P[14] to reassortment events. For example, NGS of two G8P[14] strains isolated from children with AGE in Italy in 2012 provided evidence of reassortment events between RVA strains from humans and sheep [47]. In a Slovenian study, an RVA strain isolated from a 1-year-old child with severe AGE in 2009 was genotyped using NGS, revealing a likely zoonotic origin of that strain (G8-P[14]-I2-R2-C2-M2-A3-N2-T6-E2-H3) [48]. In another study in Hungary, genome analysis of a G8P[14] isolate from a 4-year-old child with AGE in 2001 using NGS revealed that more than one reassortment event had occurred in this strain (G8-P[14]-I2-R2-C2-M2-A11-N2-T6-E2-H3) [49]. In Honduras, NGS of an RVA G10P[14] strain isolated from a 30-month-old child with severe AGE also revealed multiple reassortment events, suggesting a zoonotic origin of this strain, and potential reservoirs were identified [50]. All of these strains were pathogenic to humans, but the specific features of the virus that lead to severe AGE remain unknown. Larger studies or meta-analyses need to be conducted to address this question.

It is known that the viral proteins VP8* (derived from VP4) and VP7 play a significant role in attachment of the virus to cellular receptors containing sialic acid (SA), histo-blood group antigens (HBGAs), or cell surface components such as sialoglycans [11, 51, 52]. During endocytosis of the RVA virion, VP4 undergoes structural changes that expose hydrophobic sites of the protein [53]. VP4 contains three highly conserved trypsin cleavage sites at arginine residues 231, 241, and 247, where it is divided into two polypeptides, VP5* and VP8*, the exact length of which depends on which site is used for cleavage [54]. Cleavage of VP4 activates the infectivity of the virus [55]. In all of our samples, these regions were found to be conserved. However, we found many amino acid variants near significant sites or within antigenic epitopes. In contrast to our results, analysis of the antigenic epitopes of two G8P[14] strains in Italy showed that the sequences were conserved [47]. Further studies using mutagenesis of these strains to investigate the role of specific variants in the pathophysiology and severity of disease should be conducted.

A limitation of our study is that we did not determine the complete genotype constellations of the P[14] strains in order to identify possible reassortment events and determine their evolutionary history based on complete genome sequences. This is the first report of unusual P[14] RVA strains detected in Greece focusing on phylogenetic analysis of a part of the VP4 gene, which suggests their potential zoonotic origin. Previous studies have shown the genotype distribution of common and less common genotypes circulating in Greek children [19, 24, 56, 57]. Continuous surveillance of the distribution of RVA genotypes and their evolution is crucial for a better understanding of this virus, the disease that it causes, and the potential need to develop new RVA vaccines.

## Conclusions

In this longitudinal study, the genotype distribution of unusual G and P rotavirus strains was investigated. The long-term implementation of the RVA vaccine as well as the COVID-19 pandemic seem to have a significant impact on the epidemiology of the virus and its genotype distribution, resulting in an increase in the circulation of unusual strains. Constant surveillance of RVA genotypes is necessary for assessing the need for the development of new vaccines and estimating the breadth of coverage of circulating RVA genotypes by existing vaccines.

## Data Availability

All relevant data are within the paper.

## References

[1] Elliott EJ (2007). Acute gastroenteritis in children. BMJ Br Med J.

[2] Rivera-Dominguez G, Ward R (2021) Pediatric Gastroenteritis. In: StatPearls 29763114

[3] Mao J-W, Yang Y-L, Shi C-C (2022). Molecular epidemiological characteristics of the virus in 96 children with acute diarrhea in Changdu of Tibet, China. Zhongguo Dang Dai Er Ke Za Zhi.

[4] Kavaliotis I, Papaevangelou V, Aggelakou V (2008). ROTASCORE Study: Epidemiological observational study of acute gastroenteritis with or without rotavirus in Greek children younger than 5 years old. Eur J Pediatr.

[5] Walker PJ, Siddell SG, Lefkowitz EJ (2019). Changes to virus taxonomy and the International Code of Virus Classification and Nomenclature ratified by the International Committee on Taxonomy of Viruses (2019). Arch Virol.

[6] Leung AKC, Kellner JD, Dele Davies H (2005). Rotavirus gastroenteritis. Adv Ther.

[7] Troeger C, Khalil IA, Rao PC (2018). Rotavirus Vaccination and the Global Burden of Rotavirus Diarrhea Among Children Younger Than 5 Years. JAMA Pediatr.

[8] Shrestha S, Thakali O, Raya S (2019). Acute gastroenteritis associated with Rotavirus A among children less than 5 years of age in Nepal. BMC Infect Dis.

[9] Cortese MM, Haber P (2021) Rotavirus; Epidemiology and Prevention of Vaccine-Preventable Diseases. Commun Educ Branch, Natl Cent Immun Respir Dis Centers Dis Control Prev 19:289–300

[10] Roth GA, Abate D, Abate KH, Abay SM (2018). Global, regional, and national age-sex-specific mortality for 282 causes of death in 195 countries and territories, 1980–2017: a systematic analysis for the Global Burden of Disease Study 2017. Lancet (London England).

[11] Suzuki H (2019). Rotavirus Replication: Gaps of Knowledge on Virus Entry and Morphogenesis. Tohoku J Exp Med.

[12] Uprety T, Wang D, Li F (2021). Recent advances in rotavirus reverse genetics and its utilization in basic research and vaccine development. Arch Virol.

[13] Ciarlet M, Schödel F (2009). Development of a rotavirus vaccine: Clinical safety, immunogenicity, and efficacy of the pentavalent rotavirus vaccine. RotaTeq® Vaccine.

[14] Sadiq A, Bostan N, Jadoon K, Aziz A (2022). Effect of rotavirus genetic diversity on vaccine impact. Rev Med Virol.

[15] Dóró R, László B, Martella V (2014). Review of global rotavirus strain prevalence data from six years post vaccine licensure surveillance: is there evidence of strain selection from vaccine pressure?. Infect Genet Evol.

[16] Matthijnssens J, Potgieter CA, Ciarlet M (2009). Are Human P[14] Rotavirus Strains the Result of Interspecies Transmissions from Sheep or Other Ungulates That Belong to the Mammalian Order Artiodactyla ?. J Virol.

[17] Jamnikar-Ciglenecki U, Kuhar U, Steyer A, Kirbis A (2017). Whole genome sequence and a phylogenetic analysis of the G8P[14] group A rotavirus strain from roe deer. BMC Vet Res.

[18] DOCUMENTS AND METHODS – European Rotavirus Network. https://www.eurorotanet.com/project-information/documents-and-methods/. Accessed 23 Feb 2022

[19] Koukou D, Grivea I, Roma E (2011). Frequency, clinical characteristics, and genotype distribution of rotavirus gastroenteritis in Greece (2007–2008). J Med Virol.

[20] Sun X, Li D, Duan Z (2021). Structural Basis of Glycan Recognition of Rotavirus. Front Mol Biosci.

[21] Zeller M, Patton JT, Heylen E (2012). Genetic analyses reveal differences in the VP7 and VP4 antigenic epitopes between human rotaviruses circulating in Belgium and rotaviruses in rotarix and RotaTeq. J Clin Microbiol.

[22] Santos ML, Florentino AO, Saeki MJ (2005). Global distribution of rotavirus serotypes/genotypes and its implication for the development and implementation of an effective rotavirus vaccine. Rev Med Virol.

[23] Omatola CA, Ogunsakin RE, Olaniran AO (2021). Prevalence, Pattern and Genetic Diversity of Rotaviruses among Children under 5 Years of Age with Acute Gastroenteritis in South Africa: A Systematic Review and Meta-Analysis. Viruses.

[24] Koukou DM, Michos A, Chatzichristou P (2022). Rotavirus epidemiology and genotype distribution in hospitalised children, Greece, 2008 to 2020: A prospective multicentre study. Eurosurveillance.

[25] Roczo-Farkas S, Thomas S, Donato CM et al (2021) Australian Rotavirus Surveillance Program: Annual Report, 2020. Commun Dis Intell 45:. 10.33321/cdi.2021.45.64 10.33321/cdi.2021.45.64 34847338

[26] Roczo-Farkas S, Thomas S, Bogdanovic-Sakran N et al (2022) Australian Rotavirus Surveillance Program: Annual Report, 2021. Commun Dis Intell 46:. 10.33321/cdi.2022.46.75 10.33321/cdi.2022.46.75 36529132

[27] Okitsu S, Khamrin P, Hikita T (2022). Changing distribution of rotavirus A genotypes circulating in Japanese children with acute gastroenteritis in outpatient clinic, 2014–2020. J Infect Public Health.

[28] Hungerford D (2020) EuroRotaNet Annual Report 2020

[29] Tavakoli Nick S, Mohebbi SR, Ghaemi A, Hosseini SM (2019). Human rotavirus in Iran; molecular epidemiology, genetic diversity and recent updates on vaccine advances - PubMed. Gastroenterol Hepatol Bed Bench.

[30] Vizzi E, Piñeros OA, Oropeza MD (2017). Human rotavirus strains circulating in Venezuela after vaccine introduction: predominance of G2P[4] and reemergence of G1P[8]. Virol J.

[31] Okitsu S, Hikita T, Thongprachum A (2018). Detection and molecular characterization of two rare G8P[14] and G3P[3] rotavirus strains collected from children with acute gastroenteritis in Japan. Infect Genet Evol.

[32] Medici MC, Tummolo F, Bonica MB (2015). Genetic diversity in three bovine-like human G8P[14] and G10P[14] rotaviruses suggests independent interspecies transmission events. J Gen Virol.

[33] Bányai K, Martella V, Molnár P (2009). Genetic heterogeneity in human G6P[14] rotavirus strains detected in Hungary suggests independent zoonotic origin. J Infect.

[34] Tam KI, Roy S, Esona MD (2014). Full genomic characterization of a novel genotype combination, G4P[14], of a human rotavirus strain from Barbados. Infect Genet Evol.

[35] Damtie D, Melku M, Tessema B, Vlasova AN (2020). Prevalence and Genetic Diversity of Rotaviruses among under-Five Children in Ethiopia: A Systematic Review and Meta-Analysis. Viruses.

[36] Fritzen JTT, Oliveira MV, Lorenzetti E (2020). Genotype constellation of a rotavirus A field strain with an uncommon G8P[11] genotype combination in a rotavirus-vaccinated dairy cattle herd. Arch Virol.

[37] Strydom A, Donato CM, Nyaga MM (2021). Genetic Characterisation of South African and Mozambican Bovine Rotaviruses Reveals a Typical Bovine-like Artiodactyl Constellation Derived through Multiple Reassortment Events. Pathog (Basel Switzerland).

[38] Mozgovoj M, Miño S, Barbieri ES (2022). GII.4 human norovirus and G8P[1] bovine-like rotavirus in oysters (Crassostrea gigas) from Argentina. Int J Food Microbiol.

[39] Ahmed NU, Khair A, Hassan J (2022). Risk factors for bovine rotavirus infection and genotyping of bovine rotavirus in diarrheic calves in Bangladesh. PLoS ONE.

[40] Sawant PM, Digraskar S, Gopalkrishna V (2020). Molecular characterization of unusual G10P[33], G6P[14] genomic constellations of group A rotavirus and evidence of zooanthroponosis in bovines. Infect Genet Evol.

[41] Silva-Sales M, Leal E, de Milagres FA (2020). Genomic constellation of human Rotavirus A strains identified in Northern Brazil: a 6-year follow-up (2010–2016). Rev Inst Med Trop Sao Paulo.

[42] Damanka SA, Dennis FE, Lartey BL (2020). Next-generation sequencing of a human-animal reassortant G6P[14] rotavirus A strain from a child hospitalized with diarrhoea. Arch Virol.

[43] Ali S, Khan S, Khan SN (2021). Molecular detection and prevalence of Rotavirus with acute gastroenteritis among the children of rural and urban areas. Braz J Biol.

[44] Shams S, Mousavi Nasab SD, Heydari H (2020). Detection and characterization of rotavirus G and P types from children with acute gastroenteritis in Qom, central Iran. Gastroenterol Hepatol from bed to bench.

[45] Shoeib A, Portocarrero DEV, Wang Y, Jiang B (2020). First isolation and whole-genome characterization of a G9P[14] rotavirus strain from a diarrheic child in Egypt. J Gen Virol.

[46] Čolić D, Krešić N, Mihaljević Ž et al (2021) A Remarkable Genetic Diversity of Rotavirus A Circulating in Red Fox Population in Croatia. Pathog (Basel, Switzerland) 10:. 10.3390/PATHOGENS10040485 10.3390/pathogens10040485 PMC8072941 33923799

[47] Delogu R, Ianiro G, Morea A (2016). Molecular characterization of two rare human G8P[14] rotavirus strains, detected in Italy in 2012. Infect Genet Evol.

[48] Steyer A, Naglič T, Jamnikar-Ciglenečki U, Kuhar U (2017). Detection and Whole-Genome Analysis of a Zoonotic G8P[14] Rotavirus Strain Isolated from a Child with Diarrhea. Genome Announc.

[49] Marton S, Dóró R, Fehér E (2017). Whole genome sequencing of a rare rotavirus from archived stool sample demonstrates independent zoonotic origin of human G8P[14] strains in Hungary. Virus Res.

[50] Quaye O, Roy S, Rungsrisuriyachai K (2018). Characterisation of a rare, reassortant human G10P[14] rotavirus strain detected in Honduras. Mem Inst Oswaldo Cruz.

[51] Arias CF, Silva-Ayala D, López S (2015). Rotavirus entry: a deep journey into the cell with several exits. J Virol.

[52] Do LP, Kaneko M, Nakagomi T (2017). Molecular epidemiology of Rotavirus A, causing acute gastroenteritis hospitalizations among children in Nha Trang, Vietnam, 2007–2008: Identification of rare G9P[19] and G10P[14] strains. J Med Virol.

[53] Salgado EN, Garcia Rodriguez B, Narayanaswamy N (2018). Visualization of Calcium Ion Loss from Rotavirus during Cell Entry. J Virol.

[54] Settembre EC, Chen JZ, Dormitzer PR (2011). Atomic model of an infectious rotavirus particle. EMBO J.

[55] Rodríguez JM, Chichón FJ, Martín-Forero E (2014). New insights into rotavirus entry machinery: stabilization of rotavirus spike conformation is independent of trypsin cleavage. PLoS Pathog.

[56] Konstantopoulos A, Tragiannidis A, Fouzas S (2013). Burden of rotavirus gastroenteritis in children < 5 years of age in Greece: Hospital-based prospective surveillance (2008–2010). BMJ Open.

[57] Kokkinos PA, Ziros PG, Monini M (2013). Rare types of rotaviruses isolated from children with acute gastroenteritis in Patras. Greece Intervirology.

